# Association of MRI findings with paraspinal muscles fat infiltration at lower lumbar levels in patients with chronic low back pain: a multicenter prospective study

**DOI:** 10.1186/s12891-024-07649-x

**Published:** 2024-07-16

**Authors:** Heyi Gu†, Jingrui Hong†, Zhongwei Wang, Jiaxin Chen, Feng Yuan, Yuanming Jiang, Yingjuan Yang, Mingbin Luo, Zhenguang Zhang, Bo He, Yilong Huang, Li Sun

**Affiliations:** 1 Department of Medical Imaging, the First Affiliated Hospital of Kunming Medical University, Kunming, China; 2 Department of Radiology, Baoshan People’s Hospital, Baoshan, China; 3 Department of Radiology, Dali Bai Autonomous Prefecture People’s Hospital, Dali, China; 4 Department of Radiology, Honghe State First People’s Hospital, Honghe, China

**Keywords:** Chronic low back pain, Quantitative MRI, Paraspinal muscles, Fatty infiltration, MRI findings

## Abstract

**Objective:**

In chronic low back pain (CLBP), the relationship between spinal pathologies and paraspinal muscles fat infiltration remains unclear. This study aims to evaluate the relationship between MRI findings and paraspinal muscles morphology and fat infiltration in CLBP patients by quantitative MRI.

**Methods:**

All the CLBP patients were enrolled from July 2021 to December 2022 in four medical institutions. The cross-sectional area (CSA) and proton density fat fraction (PDFF) of the multifidus (MF) and erector spinae (ES) muscles at the central level of the L4/5 and L5/S1 intervertebral discs were measured. MRI findings included degenerative lumbar spondylolisthesis (DLS), intervertebral disc degeneration (IVDD), facet arthrosis, disc bulge or herniation, and disease duration. The relationship between MRI findings and the paraspinal muscles PDFF and CSA in CLBP patients was analyzed.

**Results:**

A total of 493 CLBP patients were included in the study (198 females, 295 males), with an average age of 45.68 ± 12.91 years. Our research indicates that the number of MRI findings are correlated with the paraspinal muscles PDFF at the L4/5 level, but is not significant. Moreover, the grading of IVDD is the primary factor influencing the paraspinal muscles PDFF at the L4-S1 level (B_ES at L4/5_=1.845, *P* < 0.05); DLS was a significant factor affecting the PDFF of MF at the L4/5 level (B = 4.774, *P* < 0.05). After including age, gender, and Body Mass Index (BMI) as control variables in the multivariable regression analysis, age has a significant positive impact on the paraspinal muscles PDFF at the L4-S1 level, with the largest AUC for ES PDFF at the L4/5 level (AUC = 0.646, cut-off value = 47.5), while males have lower PDFF compared to females. BMI has a positive impact on the ES PDFF only at the L4/5 level (AUC = 0.559, cut-off value = 24.535).

**Conclusion:**

The degree of paraspinal muscles fat infiltration in CLBP patients is related to the cumulative or synergistic effects of multiple factors, especially at the L4/L5 level. Although age and BMI are important factors affecting the degree of paraspinal muscles PDFF in CLBP patients, their diagnostic efficacy is moderate.

**Supplementary Information:**

The online version contains supplementary material available at 10.1186/s12891-024-07649-x.

## Introduction

Low back pain (LBP) has now become the leading cause of disability worldwide [1, 2]. Chronic low back pain (CLBP) accounts for approximately 23% of LBP cases [2], which is closely related to spinal stability imbalance [3]. The intervertebral discs primarily bear the vertical load on the lumbar spine. The good functional status of the paraspinal muscles is crucial for maintaining spinal structural stability [4, 5]. Muscle atrophy and fat substitution in the paraspinal muscles are major features of muscle remodeling in CLBP patients, and fat infiltration may exacerbate CLBP [6–9]. Therefore, quantifying paraspinal muscles fat infiltration has significant value in preventing the recurrence of CLBP.

Imaging parameters for evaluating paraspinal muscles mainly include muscle cross-sectional area (CSA) and the degree of fat infiltration [7, 9–15]. In recent years, the proton density fat fraction (PDFF) can be obtained with high resolution and accuracy through available Iterative Decomposition of water and fat with Echo Asymmetry and Least Square Estimation (IDEAL-IQ) [16], which aids in accurately quantifying the fat content in the paraspinal muscles, especially the intramuscular fat [6, 7, 12, 17]. This helps to further explore the relationship between lumbar spinal lesions and the paraspinal muscles in patients with CLBP.

The diversity and complexity of etiology limit the prevention and treatment strategies of CLBP. It is crucial to elucidate the interrelation between changes in paraspinal muscles and the common etiologies seen in patients with CLBP. Intervertebral disc degeneration (IVDD) is usually considered the primary cause of CLBP, especially at the L4-S1 level [16, 18]. IVDD is the basis of various clinical spine diseases [18, 19]. Research has shown that fatty infiltration of the paraspinal muscles is closely related to severe pain or functional disorders, as well as abnormal lumbar structures [20]. However, the factors influencing the rise in paraspinal muscles fatty infiltration or muscle atrophy, as well as the relationship mechanism of paraspinal muscles between fatty infiltration and spinal diseases, are still subjects of ongoing research. This includes conditions such as IVDD [6, 8, 9, 11, 12, 14, 20–22], disc herniation [7, 9, 11, 23], degenerative lumbar spondylolisthesis (DLS) [10, 24], facet joint disease [11, 15]and spinal stenosis [13, 23, 25]. There is currently still controversy over the relationship between different etiologies or disease durations and paraspinal muscles fat infiltration in CLBP patients. Currently, those studies still have some questions: Are these associations most significant when each factor is in isolation, whether the different durations of CLBP affect these associations? Whether the severity or combination of different factors or MRI-identified pathologies also affect these associations?

Therefore, the main purpose of our study was to use a novel quantitative MRI to evaluate paraspinal muscles in the posterior column, specifically the multifidus(MF) and the erector spinae(ES), and to explore the cross-sectional associations between different spinal pathologies (Intervertebral disc and facet-related lumbar MRI findings), both individually and in combination, with paraspinal muscles fat infiltration, simultaneously explore the correlation between different disease durations and these muscles fat infiltration in CLBP patients. If we can identify, or begin to rule out, any association between degenerative lumbar diseases and paraspinal muscles, it might help us better understand how, or whether, these contribute to the association with CLBP.

## Materials and methods

### Study subjects

In this study, 493 CLBP patients from four medical institutions were recruited from July 2021 to December 2022. The study was approved by the institutional review board and followed the ethical standards of the 1964 Declaration of Helsinki. Informed consent was obtained from all patients. General information and the disease duration of the patients were collected. Inclusion criteria: (1) CLBP patients with disease duration ≥ 3 months; (2) Age range from 14 to 80 years. Exclusion criteria are as follows: (1) Contraindications to MR examination and those who cannot cooperate with scanning; (2) Visceral origin of back pain (e.g., urolithiasis); (3) Spinal trauma, tumors, infections, surgeries, etc.; (4) Musculoskeletal diseases and similar family history; (5) Pregnancy; (6) Athletes or regular fitness enthusiasts; (7) Treatment within the last 3 days before scanning; (8) Diabetes and other chronic diseases.

### MRI data collection

All participating medical institutions used a 3.0T MRI scanner (MR750w, GE Healthcare, Waukesha, USA). A 24-channel phased-array spine coil was used for lumbar scanning. The scanning parameters of all centers are the same. During scanning, to reduce respiratory motion artifacts, an abdominal pressure band with appropriate pressure was applied to the subject’s abdomen. Specific scanning parameters are detailed in Table 1.

**Table 1:** Parameters of the acquired sequences

Sequence	TR(ms)	ST(mm)	SL(mm)	FOV(cm^2^)	ETL	bandwidth(kHz)	NEX	SN
Sagittal T1WI	378	4	1	32 × 32	3	41.67	3	15
Sagittal T2WI	2820	4	1	32 × 32	19	41.67	2	15
Axial T2WI	2633	3	0.5	22 × 22	18	50	4	15
IDEAL-IQ	13.9	4	-	24 × 24	3	83.33	3	24

TE: echo time; TR: time of repetition; ST: slice thickness; SL: slice increment; FOV: field of view; NEX: number of excitation; SN: slice number

### Image analysis

**Fig. 1:**
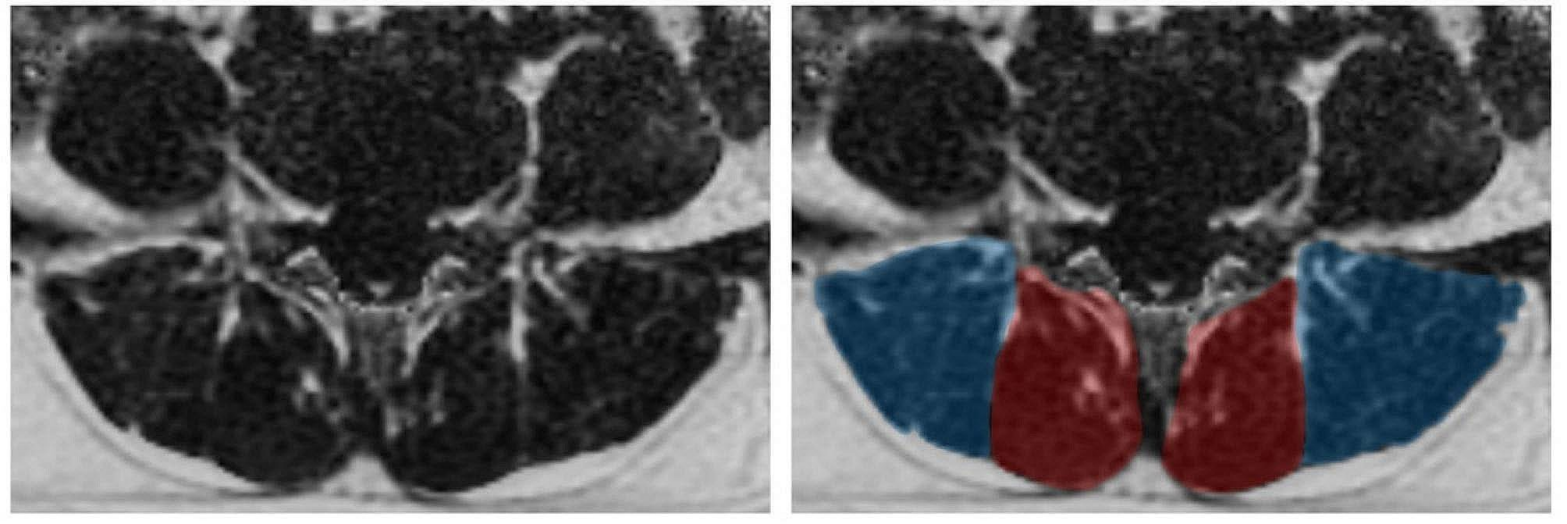
The axial position of L4/5 lumbar disc based on IDEAL-IQ in MRI (**A**)PDFF maps of paraspinal muscles; (**B**) manual segmentation of paraspinal muscles, MF (red) and ES (blue)

All raw data were transferred to the Advantage Workstation 4.6 (GE Healthcare) for processing. On regular sequences, two experienced readers blindly assessed different MRI findings at the L4-S1 level. DLS, IVDD, facet arthrosis, disc bulging, and disc protrusion were evaluated according to the criteria referenced in studies [26–29]. In case of discrepancies, the two radiologist readers discussed to reach a consensus. This study also adopted the method of Hancock [30] et al. to perform a combined analysis of MRI findings to assess the impact when multiple degenerative changes coexist in the same participant. For the measurement of CSA and PDFF of the paraspinal muscles, the central level of the L4/5 and L5/S1 intervertebral discs were studied. Both readers outlined the boundaries of the left and right MF and ES on the fat fraction map of the IDEAL-IQ sequence and the axial T2WI (Fig. 1). The average PDFF of MF and ES was obtained on the IDEAL-IQ sequence for each level, and the average PDFF of the four muscle blocks on each level was calculated. Similarly, the CSA images of MF and ES were drawn on the axial T2WI, obtaining the CSA of MF, ES, and the paraspinal musculature (PSM, MF + ES). Randomly select a portion of the CSA and PDFF data for repeatability testing, and after a certain interval, the same reader measures again, unaware of the previous measurement results.

### Statistical analysis

Statistical analysis was performed using SPSS 26.0 software (IBM Corp, Armonk, NY, USA). The Kolmogorov-Smirnov test was used for normality testing. Continuous variables are expressed as mean ± standard deviation, and categorical variables are expressed as counts and percentages. Disease duration was divided into two groups based on the median (one group ≤ 2 years, the other group > 2 years). In the comparison of different grading levels of the same MRI findings with the CSA and PDFF of the paraspinal muscles, the Mann-Whitney U test was used between the two groups, and the Kruskal-Wallis test was used among the three groups; the correlation between DLS and disease duration with the CSA and PDFF of the paraspinal muscles was analyzed using the point biserial correlation analysis; the correlation between IVDD, Facet arthrosis, the number of MRI findings, and the CSA and PDFF of the paraspinal muscles were analyzed using the Spearman correlation analysis. Additionally, we conducted univariate and multivariable linear regression analysis, incorporating age, gender, and BMI as control variables to explore the independent factors influencing paraspinal muscles fat infiltration. In the regression analysis, DLS was treated as a binary variable (presence or absence). IVDD was analyzed as ordinal variables based on its grading scales. For types of disc lesions, “normal” was defined as 0, while “disc bulge” and “herniation” were defined as 1 in the regression analysis. Subsequently, PDFF was dichotomized based on the median to construct a binary logistic regression model. The diagnostic performance of age and BMI on the increase in PDFF of the MF, ES, and paraspinal muscles at the L4/5 and L5/S1 levels was evaluated using Receiver Operating Characteristic (ROC) curves and the area under the curve (AUC). Finally, the intra-class correlation coefficient (ICC) was calculated to estimate the consistency of the measurements within observers.

## Results

### Patient characteristics

**Fig. 2:**
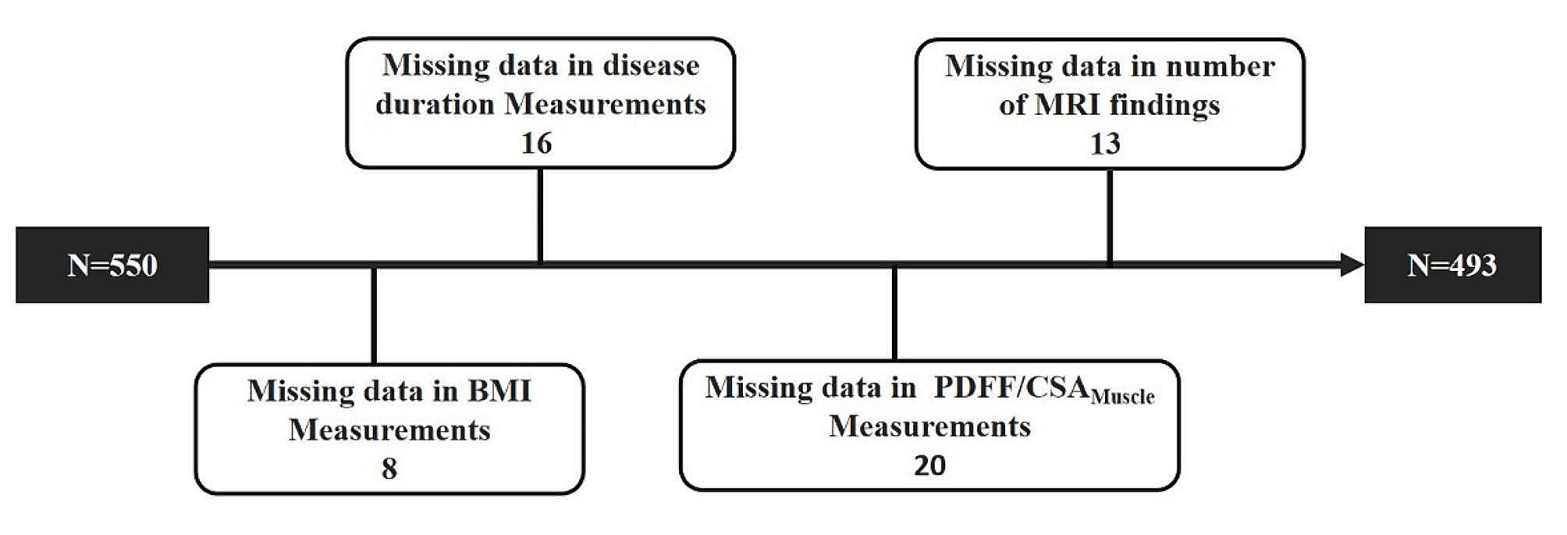
Flow diagram of the patients

A total of 493 CLBP patients were included in this study (198 females and 295 males), with an average age of 45.68 ± 12.91 years and a BMI of 22.76 ± 2.72 kg/m^2^, as shown in Table 2; Fig. 2.

**Table 2:** Demographics and characteristics of the patients

Variables(*n* = 493)		Mean ± SD	Min–Max
Sex, n(%)	Female	198(40.2)	
	Male	295(59.8)	
Age(year)		45.68 ± 12.91	14.00-78.00
BMI(kg/m^2^)		22.76 ± 2.72	16.65-37.04
Disease duration(year)		3.74 ± 4.49	0.25-30.00
L4/5			
CSA(mm^2^)	MF	841.56 ± 162.71	445.80-1416.05
	ES	1434.09 ± 359.8	579.90-3378.00
	PSM	2275.65 ± 443.08	1328-4522.00
PDFF(%)	MF	17.38 ± 7.17	4.20-48.55
	ES	17.68 ± 7.25	3.75-44.90
	PSM	17.53 ± 6.61	4.60-43.33
L5/S1			
CSA(mm^2^)	MF	1020.83 ± 184.57	499.75-1664.50
	ES	1009.38 ± 375.26	281.50-2697.90
	PSM	2030.21 ± 465.21	900.60-3942.00
PDFF(%)	MF	20.03 ± 8.08	4.00-56.04
	ES	29.33 ± 9.98	6.30-56.87
	PSM	24.68 ± 8.32	6.90-51.55

Values are expressed as numbers (%), mean ± SD, or minimum-maximum

SD: Standard Deviation; BMI: body mass index; CSA: cross-sectional area; PDFF: proton-density fat fraction; MF: multifidus; ES: erector spinae; PSM: paraspinal musculature

### Comparison of different MRI findings of CLBP patients at the L4/5 level with the CSA and PDFF of the paraspinal muscles

The intra-observer consistency for CSA and PDFF was good (CSA: ICC = 0.906, PDFF value: ICC = 0.875, *P* < 0.05). Table 3 shows the differences in different MRI findings at the L4/5 level for CLBP patients regarding the CSA and PDFF of the paraspinal muscles. DLS has a statistically significant relationship with the PDFF of MF and PSM(*p* < 0.05). The grading of IVDD and the number of MRI findings have a significant relationship with the PDFF of paraspinal muscles (*p* < 0.05). The higher the grading of IVDD or the more MRI findings, the higher the PDFF of the paraspinal muscles. The type of intervertebral disc lesion has a statistically significant relationship with the PDFF of ES and PSM (*p* < 0.05). The disease duration has a statistically significant relationship with the PDFF of MF (*p* < 0.05). However, the MRI findings mentioned above did not show statistical significance with the CSA of paraspinal muscles (*p* > 0.05). The grading of Facet arthrosis has no statistical significance with the PDFF of paraspinal muscles (*p* > 0.05), only exhibiting statistical significance with the CSA of ES (*p* < 0.05).

**Table 3:** Differences in the parameter between different MRI findings in CLBP patients at the L4/5 level

Variables	N(%)	CSA			PDFF		
	total: 493	MF	ES	PSM	MF	ES	PSM
DLS							
absent	478(97.0)	827.25(727.08,934.75)	1395.25(1168.10,1623.23)	2207.50(1954.25,2546.08)	15.93(12.15,21.06)	16.38(12.14,22.16)	16.19(12.50,21.26)
present	15(3.0)	817.50(683.00,924.00)	1418.50(1194.00,1517.50)	2242.00(1911.30,2411.00)	21.55(15.65,25.5)	20.65(13.85,25.10)	21.75(15.15,25.30)
*P*		0.66	0.93	0.69	**0.01**	0.12	**0.03**
IVDD							
Grade II	144(29.2)	811.48(724.63,916.75)	1368.13(1120.38,1658.88)	2166.28(1883.75,2595.25)	14.48(11.18,20.65)	14.53(9.78,18.70)	14.53(10.98,19.23)
Grade III	233(47.3)	832.50(733.50,950.05)	1402.80(1181.00,1606.75)	2256.00(2002.00,2540.20)	16.15(12.30,21.68)	18.25(12.93,22.75)	17.23(13.34,21.63)
Grade IV	102(20.7)	843.08(719.38,934.69)	1414.45(1186.75,1689.19)	2242.50(1953.50,2561.09)	16.30(13.51,20.69)	16.00(12.98,21.84)	16.34(13.96,21.88)
Grade V	14(2.8)	767.03(725.63,882.66)	1217.25(1166.00,1354.00)	1983.25(1876.63,2231.88)	23.09(18.91,33.54)	23.90(21.02,29.20)	24.59(20.58,31.04)
*P*		0.35	0.28	0.13	**< 0.001**	**< 0.001**	**< 0.001**
Facet arthrosis							
normal	5(1.0)	751.30(714.23,915.83)	1105.75(1085.83,1244.55)	1916.30(1842.98,2087.83)	12.75(11.03,18.73)	13.75(10.03,16.3)	13.88(10.53,17.21)
Grade I	444(90.1)	826.28(728.45,934.00)	1403.25(1175.61,1659.88)	2240.75(1962.20,2578.38)	15.90(12.16,21.33)	16.35(12.11,22.31)	16.05(12.55,21.29)
Grade II	44(8.9)	838.25(721.71,928.50)	1323.25(1098.05,1509.36)	2174.58(1905.88,2384.85)	18.88(14.50,21.66)	19.03(13.41,23.16)	18.50(14.45,22.98)
*P*		0.80	**0.02**	0.06	0.09	0.15	0.07
The types of disc lesions							
normal	185(37.7)	816.23(731.13,923.21)	1353.00(1119.09,1623.23)	2156.60(1909.63,2579.75)	15.38(11.61,20.85)	14.98(10.99,20.88)	15.01(11.71,20.23)
bulge	215(43.6)	814.00(721.00,932.00)	1401.50(1197.00,1603.50)	2242.00(1952.00,2491.50)	17.75(13.00,22.40)	18.25(12.95,22.85)	17.38(14.08,22.75)
herniation	92(18.7)	858.85(749.25,988.16)	1418.20(1175.50,1693.78)	2217.75(2009.00,2625.00)	15.83(12.21,20.18)	15.98(13.15,22.74)	16.27(12.25,20.96)
*P*		0.24	0.33	0.31	0.05	**< 0.001**	**< 0.001**
Disease duration							
≤ 2	266(54.0)	826.53(726.71,942.25)	1416.00(1177.91,1652.41)	2247.95(1963.81,2588.54)	15.30(11.75,20.81)	15.68(11.65,22.53)	15.88(11.75,21.11)
> 2	227(46.0)	829.55(726.50,922.00)	1372.50(1151.65,1599.00)	2135.00(1948.00,2502.00)	17.25(12.95,21.65)	17.00(12.50,21.95)	16.98(13.58,21.73)
*P*		0.78	0.12	0.15	**0.03**	0.28	0.06
The number of MRI findings							
1	4(8.0)	737.90(709.09,810.10)	1128.75(1091.39,1290.95)	1896.28(1826.34,2045.56)	12.10(10.81,16.13)	13.43(9.11,16.95)	12.77(9.96,16.54)
2	183(37.1)	817.50(732.50,927.00)	1366.95(1120.00,1623.90)	2157.70(1921.50,2585.00)	15.60(11.70,20.85)	14.95(11.00,20.95)	15.20(11.78,20.38)
3	293(59.4)	838.00(725.50,943.10)	1402.80(1188.50,1639.53)	2245.50(1986.35,2522.75)	16.25(12.45,21.38)	17.50(12.93,22.73)	16.95(13.56,21.78)
4	13(2.6)	817.5(702.00,918.50)	1418.50(1211.58,1500.00)	2242.00(1939.15,2408.75)	21.55(15.80,26.58)	20.65(13.55,25.70)	22.13(15.10,28.10)
*P*		0.58	0.18	0.17	**0.01**	**< 0.001**	**< 0.001**

MF: multifidus; ES: erector spinae; PSM: paraspinal musculature; DLS: Degenerative lumbar spondylolisthesis; IVDD: Intervertebral disc degeneration;

Boldface indicates *P* < 0.05

### Comparison of MRI findings in CLBP patients at the L5/S1 level with the CSA and PDFF of paraspinal muscles

Table 4 shows the differences in different MRI findings at the L5/S1 level for CLBP patients regarding the CSA and PDFF of the paraspinal muscles. DLS has a statistically significant relationship with the CSA of MF (*p* < 0.05). Compared to the non-DLS group, the DLS group has a significantly reduced CSA of MF. DLS has no statistically significant relationship with the CSA of ES, PSM, and the PDFF of paraspinal muscles (*p* > 0.05). The grading of IVDD and disease duration have a statistically significant relationship with the PDFF of paraspinal muscles (*p* < 0.05). The more severe the IVDD and the longer the disease duration, the higher the PDFF of the paraspinal muscles. However, the grading of IVDD and disease duration have no statistically significant relationship with the CSA of paraspinal muscles (*p* > 0.05). The grading of Facet arthrosis, type of intervertebral disc lesion, and the number of MRI findings have no statistically significant relationship with the CSA and PDFF of paraspinal muscles (*p* > 0.05).

**Table 4:** Differences in the parameter between different MRI findings in CLBP patients at the L5/S1 level

Variables	*N*(%)	CSA			PDFF		
	total: 493	MF	ES	PSM	MF	ES	PSM
DLS							
absent	484(98.2)	1002.90(903.30,1137.21)	962.83(765.00,1198.84)	1965.88(1729.21,2299.94)	19.35(14.15,24.59)	28.75(21.46,35.98)	24.04(18.14,30.43)
present	9(1.8)	839.50(756.25,992.75)	1142.55(586.25,1697.50)	1976.35(1517.50,2795.00)	19.75(16.05,27.45)	24.2(21.03,33.65)	21.5(18.13,30.42)
*P*		**0.02**	0.33	0.92	0.58	0.4	0.79
IVDD							
Grade II	123(24.9)	1002.30(913.00,1138.00)	956.50(798.95,1133.00)	1961.70(1767.00,2250.80)	18.05(12.8,23.9)	26.70(20.35,36.10)	22.23(17.35,27.95)
Grade III	205(41.6)	1011.50(902.25,1141.25)	977.00(742.00,1237.25)	1988.50(1742.55,2317.93)	19.6(14.33,24.88)	28.95(21.75,35.130)	24.13(18.14,30.43)
Grade IV	140(28.4)	1000.43(882.25,1134.99)	971.25(767.74,1211.13)	1934.33(1698.79,2323.00)	19.9(14.80,24.55)	28.90(21.53,35.54)	24.72(18.01,30.27)
Grade V	25(5.1)	937.90(872.00,1053.00)	835.00(643.50,1189.80)	1871.50(1571.5,2116.68)	24.25(19.23,29.30)	36.35(26.90,44.15)	30.15(22.95,38.12)
*P*		0.4	0.85	0.52	**0.01**	**0.03**	**0.01**
Facet arthrosis							
normal	3(0.6)	1006.50(996.38, 1017.90)	1138.80(879.58, 1213.00)	2168.10(1897.48, 2220.78)	20.95(20.33, 22.68)	30.45(24.78, 32.40)	25.70(22.55, 27.54)
Grade I	448(90.9)	1001.50(900.75,1140.00)	966.00(759.13,1203.16)	1970.98(1721.38,2307.63)	19.30(13.98,24.75)	28.58(21.41,35.68)	23.90(18.01,30.43)
Grade II	42(8.5)	987.80(878.98,1091.98)	934.50(799.76,1179.10)	1946.23(1753.03,2170.51)	20.83(16.38,24.84)	29.18(23.19,40.80)	24.81(19.75,31.60)
*P* ^*a*^		0.3	0.73	0.46	0.34	0.35	0.33
The types of disc lesions							
normal	192(38.9)	1004.50(899.59,1145.63)	971.25(772.76,1185.04)	1975.85(1737.58,2289.25)	19.18(14.10,24.91)	29.38(22.26,35.98)	24.09(18.63,30.43)
bulge	107(40.0)	993.00(881.38,1098.00)	975.50(736.95,1200.58)	1950.20(1713.58,2226.75)	19.75(14.33,24.23)	28.65(21.53,36.15)	23.5(17.97,30.89)
herniation	104(21.1)	1023.78(924.21,1172.88)	914.58(771.88,1269.01)	1944.33(1714.13,2395.16)	19.58(13.70,25.34)	27.73(21.16,34.68)	24.12(17.91,29.57)
*P*		0.15	0.97	0.65	0.98	0.46	0.73
Disease duration							
≤ 2	266(54.0)	1006.73(907.43,1138.24)	971.45(769.39,1229.11)	1979.55(1738.85,2324.63)	18.53(13.24,24.15)	27.58(20.20,35.01)	23.06(17.19,29.15)
>2	227(46.0)	996.00(886.50,1126.00)	956.50(751.00,1162.00)	1950.20(1716.00,2267.00)	20.2(15.30,25.55)	29.4(23.25,37.6)	25.03(19.83,31.1)
*P*		0.57	0.25	0.23	**0.02**	**0.01**	**0.01**
The number of MRI findings							
1	3(0.6)	1006.50(996.38, 1017.90)	1138.80(879.58, 1213)	2168.10(1897.48, 2220.78)	20.95(20.33, 22.68)	30.45(24.78, 32.40)	25.70(22.55, 27.54)
2	189(38.3)	1004.00(898.73,1149.00)	970.00(775.18,1183.03)	1975.35(1738.65,2293.33)	18.95(14.00,24.98)	29.20(22.38,36.08)	24.05(18.42,30.47)
3	293(59.4)	1000.85(903.60,1121.35)	952.50(762.75,1212.75)	1950.20(1718.25,2309.68)	19.60(14.15,24.48)	28.30(21.30,35.93)	23.98(17.94,30.42)
4	8(1.6)	851.00(739.13,996.38)	1229.50(586.13,1799.75)	1998.00(1483.00,2978.50)	20.48(15.20,28.20)	23.88(20.46,34.60)	22.87(16.71,31.43)
*P* ^*b*^		0.11	0.74	0.91	0.81	0.48	0.82

MF: multifidus; ES: erector spinae; PSM: paraspinal musculature; DLS: Degenerative lumbar spondylolisthesis; IVDD: Intervertebral disc degeneration;

Boldface indicates *P* < 0.05;

a, for facet arthritis, comparisons are only made between grades I and II; b, for the number of MRI findings, comparisons are only made between 2 to 4

### Relationship between different MRI findings of CLBP patients and the CSA and PDFF of paraspinal muscles

**Fig. 3:**
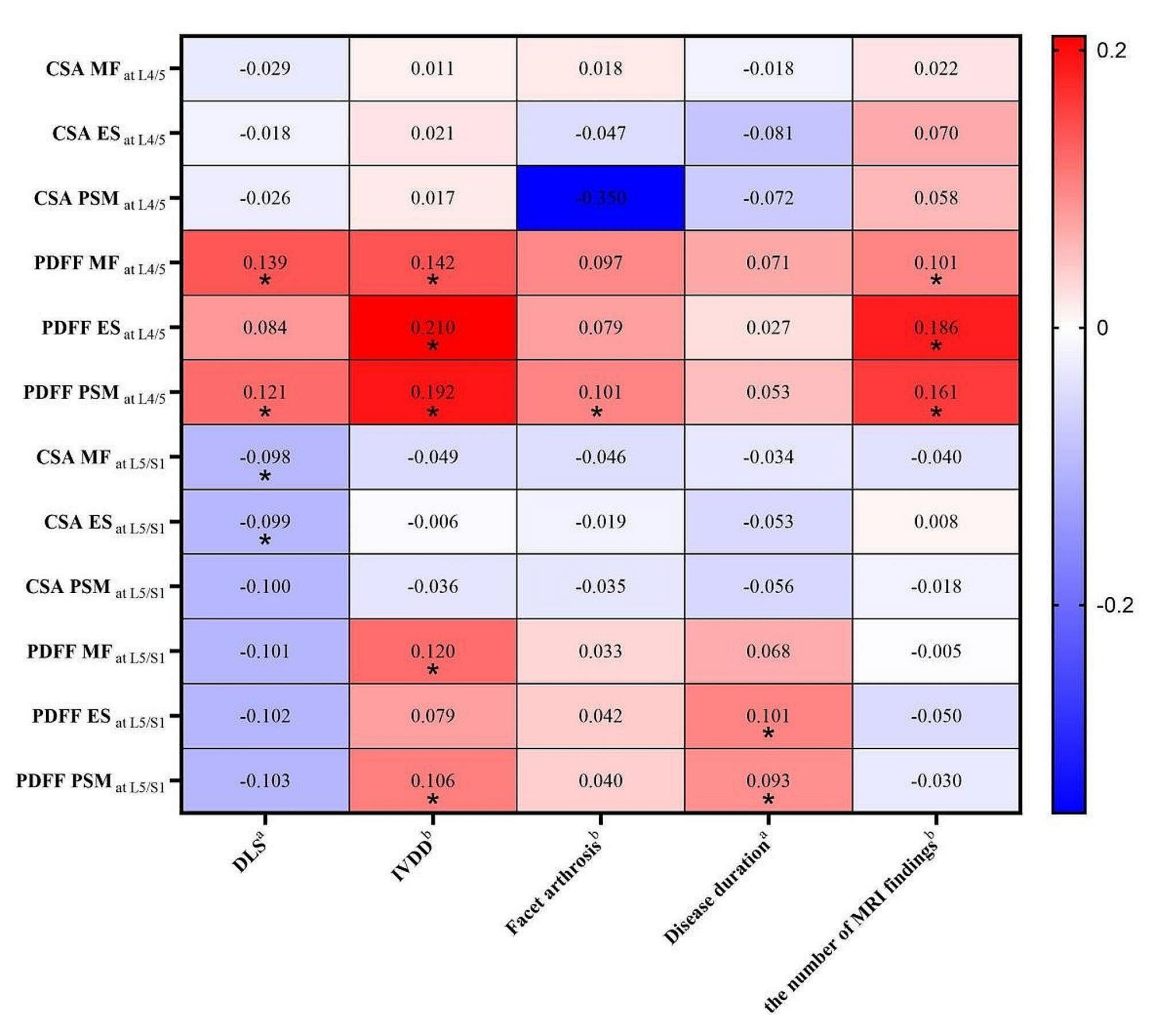
Heatmap showing the r-index between the parameters between different MRI findings in CLBP patients. For each tile, the darker the color, the stronger the correlation. a indicates point biserial correlation analysis, and b indicates Spearman correlation analysis.*, indicates *P* < 0.05; MF: multifidus; ES: erector spinae; PSM: paraspinal musculature; DLS: Degenerative lumbar spondylolisthesis; IVDD: Intervertebral disc degeneration

Figure 3 shows the relationship between DLS, IVDD, facet arthrosis, disease duration, and the CSA and PDFF of paraspinal muscles at the L4/5 and L5/S1 levels in CLBP patients. The correlation analysis results show that the grading of IVDD has a positive but not significant correlation with the PDFF of MF, PSM at the L4-S1 level, and the PDFF of ES at the L4/5 level (r_max_=0.21, *P* < 0.05). Facet arthrosis only has a weak positive correlation with the PDFF of PSM at the L4/5 level (r = 0.101, *p* < 0.05). DLS only has a weak positive correlation with the PDFF of MF and PSM at the L4/5 level (r_MF_=0.139, r_PSM_=0.121, *P* < 0.05). The number of MRI findings at the L4/5 level has a weak positive correlation with the PDFF of MF, ES, and PSM (r_MF_=0.101, r_ES_=0.186, r_PSM_=0.161, *P* < 0.05), but is not related to the PDFF of the corresponding muscles at the L5/S1 level (*P* > 0.05).

### Linear regression of different MRI findings of CLBP patients and the PDFF of paraspinal muscles

**Fig. 4:**
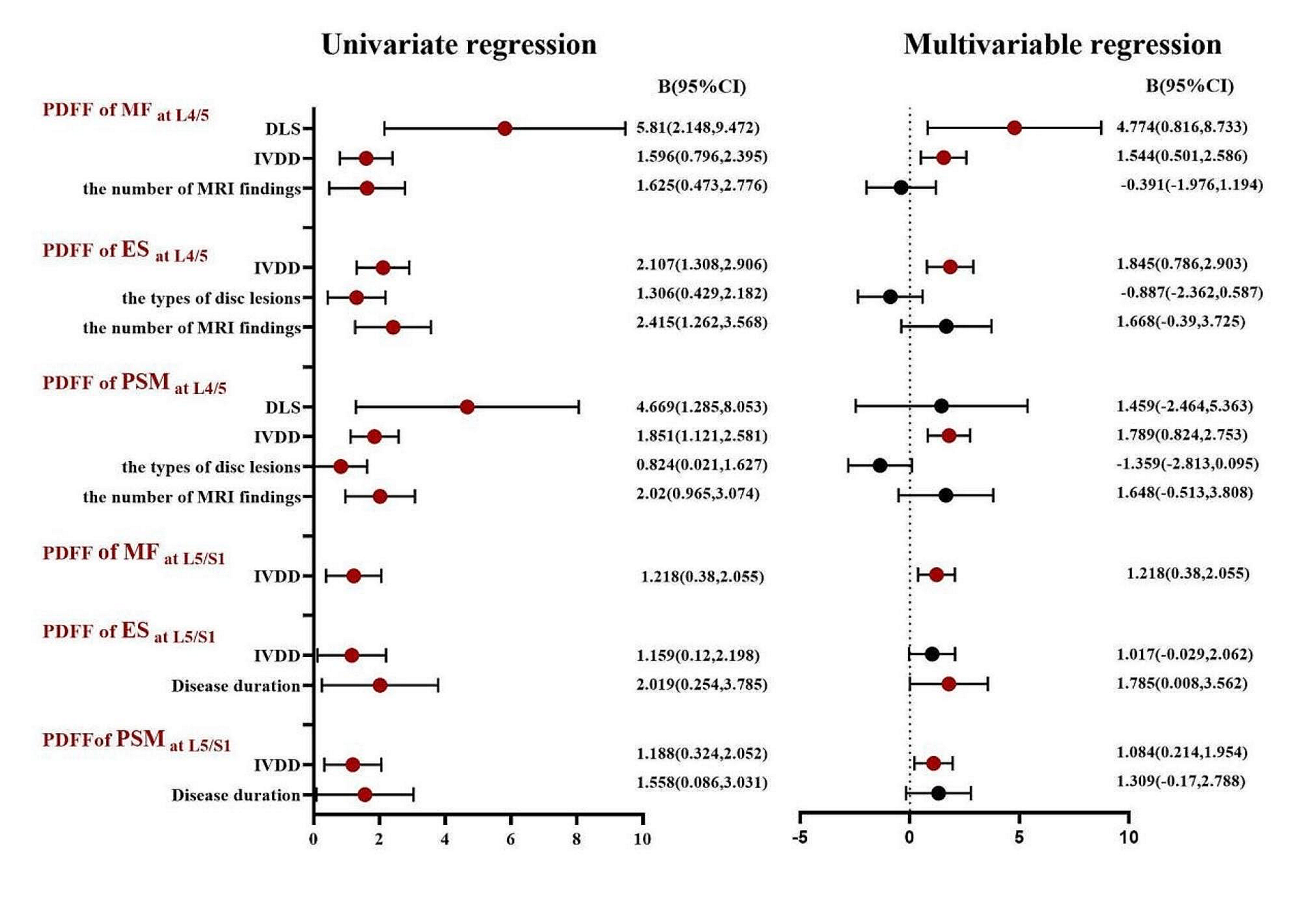
Linear regression analysis between different MRI findings and paraspinal muscles PDFF. Red indicates *p* < 0.05, and black indicates *p* > 0.05; MF: multifidus; ES: erector spinae; PSM: paraspinal musculature; DLS: Degenerative lumbar spondylolisthesis; IVDD: Intervertebral disc degeneration

Supplementary Tables 1 and Fig. 4 show the linear regression analysis of the PDFF of paraspinal muscles at the L4/5 and L5/S1 levels. At the L4/5 level, univariate linear regression analysis shows that the grading of IVDD, and the number of MRI findings all have an impact on the PDFF of MF, ES, and PSM (*P* < 0.05). After including these factors in the multivariable analysis, the results show that the grading of IVDD is a significant influencing factor for the PDFF of PSM and ES (B_ES_=1.845, B_PSM_=1.789, *p* < 0.05), and DLS is a significant influencing factor for the fat infiltration of MF (B = 4.774, *p* < 0.05). However, after including age, gender, and BMI as control variables in the analysis, the impact of DLS and the grading of IVDD on the PDFF of paraspinal muscles is no longer statistically significant (*P* > 0.05). At the L5/S1 level, univariate linear regression analysis shows that the grading of IVDD and disease duration are factors influencing the PDFF of paraspinal muscles (*p* < 0.05). After including these factors in the multivariable analysis, the results show that disease duration only has a statistically significant impact on the PDFF of ES (*p* < 0.05), while the grading of IVDD is an independent factor influencing the PDFF of MF and PSM (*p* < 0.05) but not ES (*P* > 0.05). Again, after including age, gender, and BMI as control variables, the impact of the grading of IVDD and disease duration on the PDFF of paraspinal muscles is no longer statistically significant (*P* > 0.05). Each multivariable linear model includes key statistics such as the F statistic, R-squared value, degrees of freedom (df), and Durbin-Watson statistic to assess the normality of residuals.(At the L4/5 level, PDFF of MF R^2^ = 0.330,F = 21.167, *P* < 0.001, df = 490, Durbin-Watson = 1.595;PDFF of ES R^2^ = 0.212, F = 11.572 ,*P* < 0.001, df = 490, Durbin-Watson = 1.663;PDFF of PSM R^2^ = 0.204 ,F = 11.052, *P* = 0.002 ,df = 489 ,Durbin-Watson = 1.515;At the L5/S1 level, PDFF of MF R^2^ = 0.483,F = 40.189, *P* < 0.001 ,df = 492 ,Durbin-Watson = 2.105;PDFF of ES R^2^ = 0.173 F = 9.010 *P* = 0.004 df = 491 Durbin-Watson = 1.545;PDFF of PSM R^2^ = 0.280 ,F = 16.711, *P* < 0.001, df = 491 ,Durbin-Watson = 1.600).

### ROC analysis of age and BMI on paraspinal muscles PDFF at L4/5 and L5/S1 levels

Binary logistic regression showed that BMI was statistically significant only for PDFF of the ES at the L4/5 level (AUC = 0.559, sensitivity = 0.276, specificity = 0.854, cut-off value = 24.535, *p* < 0.05). ROC analysis of age with PDFF of the MF, ES, and PSM at the L4/5 and L5/S1 levels showed that age had the highest AUC for ES PDFF at the L4/5 level (AUC = 0.646, sensitivity = 0.622, specificity = 0.619, cut-off value = 47.5, *p* < 0.001). Detailed analysis results are presented in Supplementary Table 2.

## Discussions

### The association between different spinal diseases from MRI and the paraspinal muscles CSA in CLBP patients

Our study evaluates the association between different MRI findings and CSA of the paraspinal muscles in patients with CLBP, the results indicated no significant association between all MRI findings (IVDD, disc bulge/herniation, facet arthrosis, DLS) and changes in the CSA of the paraspinal muscles, which is consistent with some other studies [14, 31, 32]. This might be due to changes in muscle tissue composition and the significant variance in the CSA of muscle among different individuals and populations. When muscle tissue atrophies, the paraspinal muscles undergo fatty infiltration. To a certain degree, because of the compensation and replacement by fatty tissue, the overall CSA of the paraspinal muscles has not changed significantly over a specific timeframe [7, 33, 34]. In the process of muscle degeneration, it might take a considerable amount of time for muscle atrophy to become apparent.

### The correlation between DLS, IVDD, and paraspinal muscles fat infiltration in CLBP patients

DLS is a condition where the upper vertebral body shifts forward or backward concerning the lower one due to degenerative elements, mainly occurring in the L4 vertebral body [14]. At present, DLS is commonly thought to be an outcome of various contributing factors, one key factor being fatty infiltration of the paraspinal muscles [35–38]. Previous research has pointed out that fat deposition within muscle tissue, insulin resistance, and mitochondrial dysfunction can lead to a decrease in skeletal muscle strength and tension. As fatty infiltration of paraspinal muscles increases, the insulin resistance and mitochondrial strength of muscle maintenance of lumbar stability will decrease, which may lead to the occurrence of DLS [39–41]. Recent research indicates that fatty infiltration of the paraspinal muscles is an independent factor influencing DLS in the L4 vertebra in asymptomatic adults, and has a high predictive value for the occurrence of DLS [10]. The results obtained by Guo et al [42]. Showed that the MF fatty infiltration in patients with degenerative lumbar instability was significantly larger than in the control group. Our study results also showed mixed results, namely, there is a weak positive correlation between DLS and PDFF of MF, with DLS having a more substantial effect on the PDFF of MF at the L4/L5 level compared to the IVDD at this level. In contrast, at the L5/S1 level, DLS is not associated with fatty infiltration of the paraspinal muscles.

The MF atrophy and fat infiltration may serve as potential risk factors for the development of degenerative spondylolisthesis [43]. The MF is the most developed and important muscle in the lumbar spine, which is a collection of paraspinal muscles with relatively smaller cross-sectional dimensions but extending almost the entire length of the spine, playing a role in lateral bending (tilting) and rotation (twisting) [44, 45]. Additionally, compared to the ES, the MF has a closer relationship with the lamina and spinous processes with more susceptibility to pathological changes and is more likely to change at the L4/L5 and L5/S1 disc levels [45]. At present, the mechanism of the relationship between MF atrophy fatty infiltration, and spinal diseases is still unclear. The two main mechanisms often mentioned are disuse and denervation [21]. We speculate that lumbar spondylolisthesis causes nerve compression, placing the MF under chronic overload and leading to disuse and denervation. This could be a key factor contributing to increased fatty infiltration and atrophy in the MF, which further reduces lumbar stability and gradually worsens the degree of slippage.

Some literature reports a significant correlation between IVDD and paraspinal muscles fatty infiltration, with the MF showing the strongest association with IVDD [7, 14]. As the Pfirrmann grading of disc degeneration increases, the fatty infiltration of the MF significantly increases [14], while the correlation between the PDFF of the ES and the Pfirrmann grading of disc degeneration is lower [7]. This is contrary to our research results, which show that the grading of IVDD has a positive but insignificant correlation with the PDFF of MF, PSM at neighboring L4-S1 disc level, and the PDFF of ES at L4/L5 level. On the other hand, certain studies argue that there is no statistically significant or weak relationship between IVDD and paraspinal muscles fatty infiltration [20, 21], which is consistent with our research results. In our study, compared with the group without disc bulge/ herniation, the disc bulge/ herniation group exhibited higher PDFF values of the ES and the PSM at the L4/L5 disc level, representing a statistically significant difference. However, our correlation analysis results showed no significant association between whether the disc was bulging/ herniation and the fatty infiltration of the adjacent ES or PSM. This inconsistency is the study by Özcan-Ekşi et al [46], which suggests patients with severe lumbar disc herniation show higher fatty infiltration of the MF and ES.

### Analysis of factors influencing paraspinal muscles fat infiltration in CLBP patients

The findings of the multivariable linear regression in our study show that IVDD impacts the fat infiltration of the MF, the ES, and the PSM. Notably, the grading of IVDD is an independent influencing factor for the fat infiltration of the PSM at the L4-S1 disc level and the ES at the L4/L5 level, DLS is a significant influencing factor for fat infiltration of the MF. Additionally, in our study, there was no association between the number of MRI findings and the CSA of paraspinal muscles in the posterior column, but there was a significant statistical difference and a weak correlation with the PDFF of MF, ES, and PSM at the L4/L5 level. As the number of MRI findings increased, the PDFF of PSM at the L4/L5 level gradually heightened, which is consistent with the results of recent research [11, 30]. Moreover, we evaluated the association between the duration of pain in CLBP patients (≤ 2 years, > 2 years) and the morphology and fat infiltration of paraspinal muscles in the posterior column. The longer the duration of pain, the more the PDFF of MF and PSM at the L4-S1 level and the PDFF of ES at the L5/S1 level increased, with statistically significant differences. Correlation analysis and multivariable linear regression results show a weak positive correlation between the duration of pain and the increase in PDFF of the ES, and this influence is only present at the L5/S1 level but is not significant. This effect disappears after adjusting for covariates. We speculate that the longer the duration of CLBP, the stronger the potential impact on the fat infiltration of the ES compared to the MF.

Previous studies have reported that age and gender affect overall muscle mass, which further affects back muscle atrophy and fat infiltration, and as age increases, fat infiltration increases [7, 47]. One study found that women have more fat infiltration in the MF and ES at the L4/L5 and L5/S1 levels, as well as men have more fat infiltration in the PSM at the L5/S1 level [46], which may be related to the decrease in muscle function due to hormone deficiency after menopause [33, 48], and the chosen level of the intervertebral disc [14]. While the LBP has been reported to have a significant correlation with BMI, the association between paraspinal muscles fat infiltration and BMI remains a matter of debate [14, 22, 33]. Therefore, after adjusting for control variables such as age, gender, and BMI, which might influence muscle fat., the impact of IVDD and DLS on paraspinal muscles fat infiltration remains unclear. This also indirectly indicates that age, gender, and BMI are important influencing factors for the degree of paraspinal muscles fat infiltration in CLBP patients. In this study, at the L4-S1 level, age has a positive impact on the increase in PDFF of paraspinal muscles, and BMI has a statistically significant positive effect on the rise in ES PDFF only at the L4/5 level, while males had a lower PDFF compared to females, which is consistent with previous research findings [7, 14, 22, 46, 47]. To evaluate the impact of age and BMI on the increase in paraspinal muscles fat infiltration, we divided PDFF into two groups based on the median and classified PDFF by age and BMI. The results showed that BMI was statistically significant only for the ES PDFF at the L4/5 level (AUC = 0.559, cut-off value = 24.535), while age had the highest AUC for ES PDFF at the L4/5 level (AUC = 0.646, cut-off value = 47.5). In summary, although age and BMI are important factors affecting the degree of paraspinal muscles fat infiltration in CLBP patients, their diagnostic efficacy in evaluating the increase in PDFF of paraspinal muscles is moderate.

Studies affirm that the stability of the spine is maintained not only by the paraspinal muscles but also by other components such as the vertebrae, intervertebral discs, facet joints, ligaments, muscles, and tendons [49, 50]. These structures, while independent, are interrelated and can compensate when any one element is damaged or degenerates. Although there is research on a causal relationship between spinal pathology and muscle atrophy in animal models, the direction of causality in humans is still unclear [8]. Thus far, there is no consensus on the causal relationship between various factors and paraspinal muscles atrophy and fat infiltration in CLBP patients, and the exact contributions of IVDD and DLS to the alterations of pain and the muscles in CLBP patients, are still undefined. It is still unclear whether intervertebral disc degeneration is significantly positively correlated with paraspinal muscles fat infiltration and whether the IVDD or DLS is the independent influencing factor for evaluating changes in paraspinal muscles quality. The causal explanation between the increase in PDFF of paraspinal muscles and lumbar diseases in patients with low back pain remains speculative.

Based on the literature review and the results of this study, we are inclined to believe that there is a complex interplay between muscle degeneration and surrounding anatomical/pathological findings as well as individual factors. The fat infiltration of paraspinal muscles in CLBP patients is influenced by the combined or synergistic effects of various factors, especially at the L4/L5 level. The relationship between IVDD and paraspinal muscles fat infiltration in the posterior column of the spine in CLBP patients may not be purely causal. Future research will explore the specific combinations of factors that significantly contribute to the overall impact on paraspinal muscles fat infiltration.

There are several limitations of this study. Currently, histopathology is considered the gold standard for quantifying fat content. Our research results were not compared with live tissue histopathology, so we cannot demonstrate a causal relationship between these factors and paraspinal muscles fat infiltration. Nevertheless, these findings emphasize the complex biomechanics between lumbar degenerative diseases, adjacent PSM, intervertebral discs, and facet arthrosis. The paraspinal muscles have a longitudinally distributed structure, but this study only selected the posterior column muscle planes corresponding to the L4/L5 and L5/S1 intervertebral discs as the levels of interest, because they are the disc levels where lumbar degenerative diseases are prone to occur. Additionally, our sample size for the prospective cross-sectional study was relatively small, although we included patients from four different medical institutions to increase sample diversity and the generalizability of the results. We regret that the relatively small sample size may have contributed to the uncertainty and lack of statistical significance in some subgroup analyses in this study, which also affected the comparative analysis between specific groups. Due to the limited sample size, this study did not group MRI findings of CLBP patients by disease duration. We speculate that the degree of paraspinal muscles atrophy and fat infiltration may change over time or pre- and post-treatment. In future studies, we will consider expanding the sample size and plan to conduct follow-up and collect high-quality longitudinal data to advance this line of research.

## Conclusion

The results of this study show that the degree of paraspinal muscles fat infiltration in CLBP patients is related to the cumulative or synergistic effects of multiple factors, especially at the L4/L5 disc level. The relationship between IVDD and paraspinal muscles fat infiltration in the posterior column of the spine in CLBP patients may not be purely causal. Although age and BMI are important factors affecting the degree of paraspinal muscles fat infiltration in CLBP patients, their diagnostic efficacy in evaluating the increase in PDFF of paraspinal muscles is moderate. In our study, with the increase in the number of imaging findings, fat infiltration of the posterior column muscles at the L4/L5 level increased, and the longer the duration of pain, the gradual increase in the ES fat infiltration at the L5/S1 level, but the correlation was weak. Whether there is a potential “dose-time-response relationship” among the three requires further study.

### Electronic supplementary material

Below is the link to the electronic supplementary material.

Supplementary Material 1

## Data Availability

Datasets can be accessed from the corresponding author upon request.

## Abbreviations

CLBP: Chronic low back pain
MRI: Magnetic resonance imaging
CSA: Cross-sectional area
PDFF: Proton density fat fraction
MF: Multifidus
ES: Erector spinae
DLS: Degenerative lumbar spondylolisthesis
IVDD: Intervertebral disc degeneration
BMI: Body Mass Index
LBP: Low back pain
PSM: Paraspinal musculature
ROC: Receiver Operating Characteristic
AUC: Area under the curve
ICC: Intra-class correlation coefficient
