# Supplementary material for: Association of MRI findings with paraspinal muscles fat infiltration at lower lumbar levels in patients with chronic low back pain: a multicenter prospective study
Source: BMC Musculoskelet Disord. 2024 Jul 16;25:549. doi: 10.1186/s12891-024-07649-x (PMC11251387; doi:10.1186/s12891-024-07649-x)
Supplement: Supplementary file 1 — Supplementary Material 1 [file 12891_2024_7649_MOESM1_ESM.docx]

**Supplementary Table 1** different MRI findings associated with paraspinal muscles PDFF in patients with CLBP by regression analyses.

| Variables | Univariate regression | | Multivariable regression | | Adjusted models* | |
| --- | --- | --- | --- | --- | --- | --- |
|  | B(95%CI) | P value | B(95%CI) | P value | B(95%CI) | P value |
| PDFF of MF at L4/5 | | | | |  |  |
| DLS | 5.81（2.148，9.472） | **0.002** | 4.774（0.816，8.733） | **0.018** | 3.344(-0.332,7.02) | 0.074 |
| IVDD | 1.596（0.796，2.395） | **<0.001** | 1.544（0.501，2.586） | **0.004** | 0.387(-0.663,1.437) | 0.469 |
| The number of MRI findings | 1.625（0.473，2.776） | **0.006** | -0.391（-1.976，1.194） | 0.628 | -1.391(-2.888,0.107) | 0.069 |
| Age | - | **-** | - | - | 0.166(0.109,0.223) | **<0.001** |
| Sex | - | **-** | - | - | -4.435(-5.631,-3.24) | **<0.001** |
| BMI | - | **-** | - | - | 0.041(-0.175,0.258) | 0.707 |
| PDFF of ES at L4/5 | | | | |  |  |
| IVDD | 2.107（1.308，2.906） | **<0.001** | 1.845（0.786，2.903） | **0.001** | 0.41(-0.673,1.493) | 0.457 |
| The types of disc lesions | 1.306（0.429，2.182） | **0.004** | -0.887（-2.362，0.587） | 0.238 | -0.138(-1.517,1.241) | 0.844 |
| The number of MRI findings | 2.415（1.262，3.568） | **<0.001** | 1.668（-0.39，3.725） | 0.112 | -0.494(-2.464,1.476) | 0.623 |
| Age | - | **-** | - | - | 0.189(0.131,0.247) | **<0.001** |
| Sex | - | **-** | - | - | -3.421(-4.618,-2.223) | **<0.001** |
| BMI | - | **-** | - | - | 0.305(0.086,0.524) | **0.006** |
| PDFF of PSM at L4/5 | | | | |  |  |
| DLS | 4.669（1.285，8.053） | **0.007** | 1.45（-2.464，5.363） | 0.467 | 1.041(-2.57,4.652) | 0.571 |
| IVDD | 1.851（1.121，2.581） | **<0.001** | 1.789（0.824，2.753） | **<0.001** | 0.488(-0.483,1.46) | 0.324 |
| The types of disc lesions | 0.824（0.021，1.627） | **0.044** | -1.359（-2.813，0.095） | 0.067 | -0.668(-2.009,0.672) | 0.328 |
| The number of MRI findings | 2.02（0.965，3.074） | **<0.001** | 1.648（-0.513，3.808） | 0.135 | -0.261(-2.298,1.775) | 0.801 |
| Age | - | **-** | - | - | 0.174(0.121,0.226) | **<0.001** |
| Sex | - | **-** | - | - | -3.935(-5.018,-2.853) | **<0.001** |
| BMI | - | **-** | - | - | 0.175(-0.021,0.371) | 0.08 |
| PDFF of MF at L5-S1 | | | | |  |  |
| IVDD | 1.218（0.38，2.055） | **0.004** | 1.218（0.38，2.055） | **0.004** | 0.488(-0.405,1.381) | 0.284 |
| Age | - | **-** | - | **-** | 0.12(0.061,0.179) | **<0.001** |
| Sex | - | **-** | - | **-** | -4.796(-6.174,-3.418) | **<0.001** |
| BMI | - | **-** | - | **-** | 0.036(-0.216,0.287) | 0.78 |
| PDFF of ES at L5-S1 |  |  |  |  |  |  |
| IVDD | 1.159（0.12，2.198） | **0.029** | 1.017(-0.029,2.062) | 0.057 | 0.1559(-0.98,1.29) | 0.788 |
| Disease duration | 2.019（0.254，3.785） | **0.025** | 1.785(0.008,3.562) | **0.049** | 1.178(-0.536,2.893) | 0.178 |
| Age | - | **-** | - | - | 0.138(0.063,0.212) | **<0.001** |
| Sex | - | **-** | - | - | -4.637(-6.385,-2.889) | **<0.001** |
| BMI | - | **-** | - | - | 0.144(-0.174,0.462) | 0.375 |
| PDFF of PSM at L5-S1 | | | | |  |  |
| IVDD | 1.188（0.324，2.052） | **0.007** | 1.084（0.214，1.954） | **0.015** | 0.314(-0.613,1.241) | 0.506 |
| Disease duration | 1.558（0.086，3.031） | **0.038** | 1.309（-0.17，2.788） | 0.083 | 0.717(-0.683,2.118) | 0.315 |
| Age | - | **-** | - | - | 0.129(0.068,0.19) | **<0.001** |
| Sex | - | **-** | - | - | -4.708(-6.135,-3.28) | **<0.001** |
| BMI | - | **-** | - | - | 0.089(-0.17,0.349) | 0.499 |

MF, multifidus; ES, erector spinae; PSM, paraspinal musculature; DLS, Degenerative lumbar spondylolisthesis; IVDD, Intervertebral disc degeneration; BMI, Body Mass Index

Boldface indicates P < 0.05;

*, indicates adjustment for age, gender, and BMI.

**Supplementary Table 2** ROC analysis of age and BMI on paraspinal muscles PDFF at L4/5 and L5/S1 levels.

| Variables | AUC(95%CI) | Sensitivity | Specificity | Cut-Off value | P value |
| --- | --- | --- | --- | --- | --- |
| PDFF of MF at L4/5 |  |  |  |  |  |
| Age | 0.625(0.576,0.675) | 0.687 | 0.563 | 45.5 | **<0.001** |
| BMI | - | - | - | - | - |
| PDFF of ES at L4/5 |  |  |  |  |  |
| Age | 0.646(0.598,0.695) | 0.622 | 0.619 | 47.5 | **<0.001** |
| BMI | 0.559(0.509,0.61) | 0.276 | 0.854 | 24.535 | **0.022** |
| PDFF of PSM at L4/5 |  |  |  |  |  |
| Age | 0.649(0.6,0.698) | 0.699 | 0.575 | 45.5 | **<0.001** |
| BMI | - | - | - | - | - |
| PDFF of MF at L5/S1 |  |  |  |  |  |
| Age | 0.612(0.562,0.662) | 0.606 | 0.603 | 47.5 | **<0.001** |
| BMI | - | - | - | - | - |
| PDFF of ES at L5/S1 |  |  |  |  |  |
| Age | 0.6(0.55,0.65) | 0.561 | 0.615 | 48.5 | **<0.001** |
| BMI | - | - | - | - | - |
| PDFF of PSM at L5/S1 |  |  |  |  |  |
| Age | 0.623(0.573,0.672) | 0.614 | 0.611 | 47.5 | **<0.001** |
| BMI | - | - | - | - | - |

MF, multifidus; ES, erector spinae; PSM, paraspinal musculature; BMI, Body Mass Index; AUC, Area Under the Curve; CI, Confidence Interval.

Boldface indicates P < 0.05.
